# Incidence, prevalence, and predictors of osteoporotic fracture in adult lung transplant recipients

**DOI:** 10.1016/j.jhlto.2024.100182

**Published:** 2024-11-20

**Authors:** Elisabeth Ng, Shanal Kumar, Eldho Paul, Daniel Bennett, Luisa Rosi, Louise Fuller, Lauren Chiu, Shoshana Sztal-Mazer, Steven Ivulich, Greg Snell, Leon A. Bach, Kathryn L. Hackman

**Affiliations:** a Department of Endocrinology and Diabetes, Alfred Health, Melbourne, Victoria, Australia; b Monash Centre for Health Research and Implementation, Faculty of Medicine, Nursing and Health Sciences, Monash University, Clayton, Victoria, Australia; c Adult Cystic Fibrosis Centre, The Prince Charles Hospital, Brisbane, Queensland, Australia; d Australian and New Zealand Intensive Care Research Centre, School of Public Health and Preventive Medicine, Monash University, Clayton, Victoria, Australia; e Department of Medicine (Alfred), Monash University, Melbourne, Victoria, Australia; f Department of Physiotherapy, Alfred Health, Melbourne, Victoria, Australia; g Department of Pharmacy, Alfred Health, Melbourne, Victoria, Australia; h Department of Respiratory Medicine, Alfred Health, Melbourne, Victoria, Australia

**Keywords:** lung transplantation, osteoporosis, fracture, bone, antiresorptive, mortality

## Abstract

**Background:**

As life expectancy following lung transplantation (LT) improves, vulnerability to glucocorticoid-induced osteoporotic fractures is increased. Our institution offers LT recipients protocolized antiresorptive therapy, with zoledronic acid (ZA) used first line.

**Methods:**

Adults who underwent LT from January 2012 to December 2018 and survived at least 6 months were retrospectively studied. Coprimary outcomes were incidence, prevalence, and predictors of osteoporotic fractures and major osteoporotic fractures post-LT.

**Results:**

Four hundred and five LT recipients (41% female, median age 59 years) had a median follow-up of 4.9 years (interquartile range 3.4-6.7). Osteoporotic fracture prevalence was 12% (*n* = 49) pre-LT and 15% (*n* = 60) post-LT. Major osteoporotic fracture post-LT occurred in 11% (*n* = 45). Antiresorptive therapy was received by 47% pre- and 89% post-LT. On multivariate analysis, risk factors for osteoporotic fracture were pre-LT osteoporotic fracture (hazard ratio (HR) 2.32 (95% confidence interval (CI) 1.09-4.96)), female sex (HR 2.08 (95% CI 1.09-3.94)), glucocorticoid use pre-LT (HR 2.08 (95% CI 1.09-3.99)), and time (months) to first ZA infusion post-LT (HR 1.04 (95% CI 1.01-1.06)). Risk factors for major osteoporotic fracture were pre-LT osteoporotic fracture, female sex, age, and time to first ZA infusion.

**Conclusion:**

LT recipients receiving protocolized antiresorptive treatment post-LT had a low incidence of osteoporotic fracture.

## Background

As survival following lung transplantation (LT) increases,^1^ long-term sequelae of immunosuppression, such as osteoporosis, are increasingly important. Most LT candidates have multiple risk factors for osteoporosis, including smoking, inactivity, low body mass index, sarcopenia, hypoxia, malnutrition, hypogonadism, hypovitaminosis D, chronic inflammation, and glucocorticoid therapy.^2^, ^3^ Pre-LT osteoporosis has been reported in up to 61% of individuals with end-stage lung disease, though contemporary data are limited.^4^ Post-LT, glucocorticoid-, and calcineurin inhibitor-containing immunosuppressive regimens accelerate bone loss, particularly in the first year.^5^, ^6^ Importantly, glucocorticoids predominantly affect trabecular bone, increasing the risk of vertebral fractures.^7^ Post-LT fractures result in pain, reduced physical function, and compromised lung function with thoracic vertebral and rib fractures.^8^

The reported prevalence of osteoporotic fractures in the first year after LT is 17% to 37% despite bone preservation therapies, but data beyond the first year are limited,^6^, ^9^, ^10^, ^11^, ^12^ and no studies have considered mortality when assessing fracture risk. This is important as 12% of patients do not survive beyond 12 months, and of those who do, 30% do not survive beyond 5 years.^13^

LT recipients require lifelong glucocorticoids but there is no international consensus regarding routine use of bone preservation therapies. A 2001 study suggested a benefit for bone preservation and fracture rate with pamidronate.^12^ In 2012, our center protocolized zoledronic acid (ZA) administration in LT candidates in anticipation of post-LT bone loss, based on local multidisciplinary team expert guidance. In the absence of Australian or international standard protocols, we sought to evaluate the efficacy and safety of our proactive approach to bone management in LT recipients.

The primary objective was to determine the incidence, prevalence, and risk factors for (i) all osteoporotic fractures and (ii) major osteoporotic fractures in a contemporary cohort of adult LT recipients. Secondary objectives were to evaluate the prevalence of nonosteoporotic fractures and treatment-related adverse events.

## Methods

### Study design

This was a retrospective study of all LT recipients aged ≥18 years who underwent single, bilateral, or heart-lung transplants at Alfred Health between January 1, 2012 and December 31, 2018. Data were censored on January 7, 2022*.* This time frame allowed at least 3 years of follow-up. Exclusion criteria were death within 6 months of LT, relocation with external follow-up, and second or subsequent LT within the study period.

### Data collection

Data obtained from the electronic medical records included (1) LT date and indication, (2) clinical characteristics (age, sex, smoking history, pre-LT glucocorticoid use, use of high-dose methylprednisolone), (3) 6-minute walk test (6MWT) pre- and post-LT^14^, (4) use of bone preservation therapies pre- and post-LT, (5) treatment-related adverse effects (atypical femoral fracture, osteonecrosis of the jaw and acute phase reactions); (6) osteoporotic fracture pre-LT, (7) date and location of post-LT fractures, and (8) date of death. ZA administration was determined with time to ZA defined from LT-date to date of first ZA dose post-LT.

Fractures were identified through systematic review of inpatient and outpatient documentation and corroborated with radiology reports. Post-LT fractures were classified as (1) osteoporotic, if they were low trauma; subdivided into major (vertebral, hip, proximal humerus, distal radius)^15^, ^16^ or other (pelvis or ribs); (2) nonosteoporotic (feet, ankles, or digits); or (3) atypical femoral fracture.

At least 10% of data were independently cross-checked for quality control. Regular meetings were conducted to reach consensus in complex cases. This study was approved by the Alfred Health Ethics Committee and designated low risk with waiver of consent due to its retrospective design (Project 79/20).

### Center-based protocol for LT management

The Alfred Hospital approach to recipient selection is based on international guidelines.^17^, ^18^ Transplantation followed standard practice, with LT recipients receiving triple immunosuppression with initial tacrolimus, azathioprine, and corticosteroids. Initial prednisolone dose was 50 mg daily, weaning to 15 mg by 3 months and 5 to 7.5 mg by 1 to 2 years.^19^ Therapy was clinically modified in the presence of significant rejection, renal impairment, or systemic sepsis. All patients were followed in Alfred Lung Transplant Multidisciplinary Clinics monthly in the first year and at least 3 monthly thereafter (including in-house physiotherapy as required). All patients were strongly encouraged to undertake pulmonary rehabilitation pre-LT and were mandated to undertake a structured, supervised exercise rehabilitation for at least 7 weeks post hospital discharge.^20^

### Antiresorptive therapy protocol

In 2012, the Department of Endocrinology at Alfred Health implemented a protocol to prevent post-LT bone loss (Supplement S1). The protocol recommends regular intravenous ZA infusions from waitlisting until prednisolone dose is <7.5 mg, with patient weight, renal function, absolute bone mineral density (BMD), change in BMD, and bone turnover makers taken into consideration. The final decision is made by the treating endocrinologist.

### Statistical analysis

Categorical variables were summarized with subject counts and percentages. Nonparametric continuous variables were expressed as medians (interquartile ranges [IQR]). Incidence and risk factors for first osteoporotic fracture and first major osteoporotic fracture were coprimary end-points. Univariate and multivariate analyses were performed using competing risk regression, with death as a competing variable. Results are reported as hazard ratios (HR) (95% confidence intervals (CI)). Univariate variables with *p* < 0.05 and those deemed clinically relevant were entered into a hierarchical regression model to identify independent risk factors. Covariates in these analyses were age, sex, pretransplantation osteoporotic fracture and glucocorticoid use, gym exit 6MWT, and underlying lung disease. Cumulative incidence of osteoporotic fracture was estimated with death as the competing event.

To test the robustness of the association between time to first ZA infusion post-LT and osteoporotic fracture, a sensitivity analysis was performed in a propensity score matched cohort using a multivariable logistic regression model that included age, gender, lung disease, transplant wait-time, and pre-LT osteoporotic fracture as predictor variables. This propensity score–matched patients who received ZA post-LT to those who did not, using 1-to-1 nearest neighborhood matching. All *p*-values are 2-sided with *p* < 0.05 indicating statistical significance. Analyses were performed using SPSS version 26 (IBM) and SAS software version 9.4 (SAS Institute, Cary, NC).

## Results

### Patient characteristics

**Figure 1:**
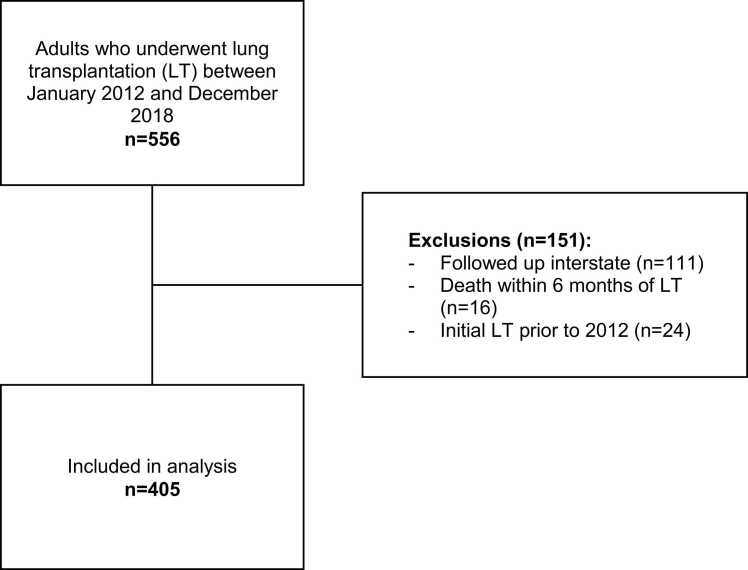
Flow diagram of lung transplantation recipients included in the study. LT, lung transplantation.

Between January 2012 and December 2018, 556 adults underwent LT, of whom 151 were excluded, leaving 405 for analysis (Figure 1). Median follow-up was 4.9 years (IQR 3.4-6.7), maximum 8 years. Survival was 96% (95% CI 93%-97%) 1 year post-LT, 83% (79%-86%) at 3 years, and 72% (67%-76%) at 5 years. Overall, 146 (36%) patients died (85 male, 61 female). Participant characteristics are presented in Table 1.

**Table 1:** Lung Transplantation Recipient Characteristics

Participant characteristics	Total *n* = 405
Age at transplantation, years	59 (51-65)
Male sex	237 (58.5%)
Exsmoker	83 (20.6%)
Body mass index, kg/m^2^	24.1 (20.5-27.4)
Glucocorticoid use pretransplant	234 (57.8%)
Reason for LT	
Obstructive lung disease	194 (46.9%)
Restrictive lung disease	114 (28.1%)
Cystic fibrosis	58 (14.3%)
Pulmonary hypertension	26 (9.8%)
Other	13 (3.2)
Transplant type	
Double lung transplant	367 (90.6%)
Single lung transplant	36 (8.9%)
Heart and lung transplant	2 (0.5%)
Waiting time, days	87 (36, 204)
5-year survival^a^	72% (95% CI 67%-76%)
6-minute walk test (6MWT), meters	
Pre-LT 6MWT (*n* = 390)	292 (191-370)
Entry to gym 6MWT (*n* = 403)	330 (240-394)
Exit gym 6MWT (*n* = 396)	523 (435-595)

Abbreviations: CI, confidence interval; LT, lung transplantation.

Continuous data are presented here as median (25-75 centile) and categorical data as *n* (%).

a Patients who died within 6 months of transplant (*n* = 16) were excluded.

### Primary outcomes

**Figure 2:**
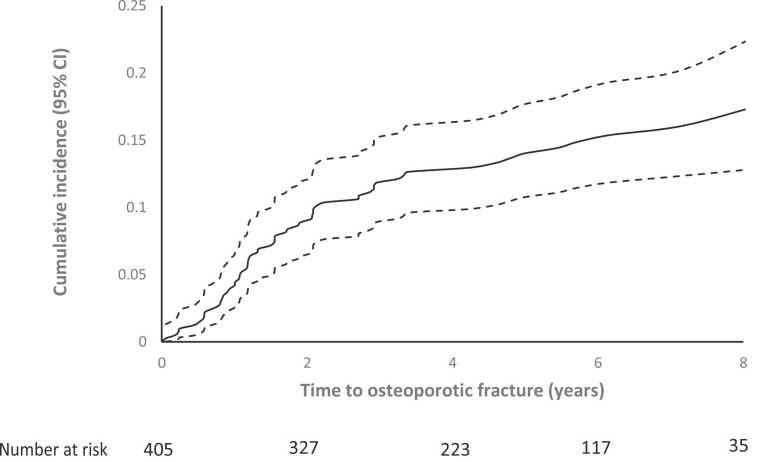
Cumulative incidence of first osteoporotic fracture over time with death as a competing event. CI, confidence interval.

**Figure 3:**
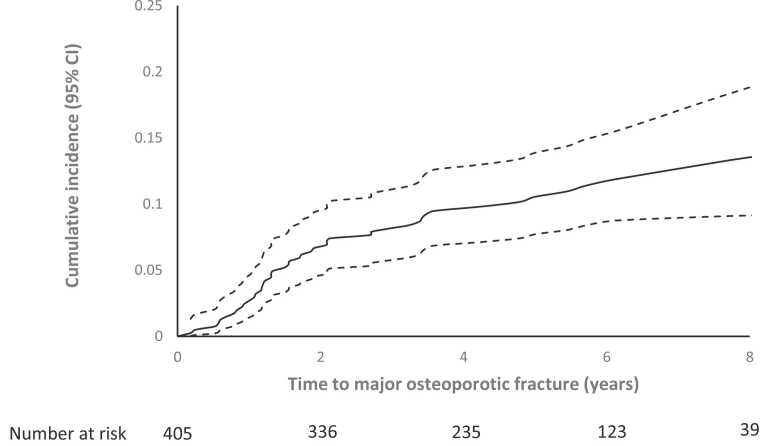
Cumulative incidence of first major osteoporotic fracture over time, with death as a competing event. CI, confidence interval.

Cumulative incidence of post-LT osteoporotic fracture was 4.0%, 11.9%, and 14.0% at 1, 3, and 5 years, respectively. For major osteoporotic fracture, the cumulative incidence was 2.5%, 8.2%, and 10.5% at 1, 3, and 5 years, respectively. Cumulative probability of first fracture, with death as a competing event, is shown in Figure 2 (all osteoporotic fractures) and Figure 3 (major osteoporotic fractures).

The prevalence of osteoporotic fracture was 23.5% (*n* = 95): 12.1% (*n* = 49) pre-LT and 14.8% (*n* = 60) post-LT. Fourteen had osteoporotic fractures pre- and post-LT. Major osteoporotic fracture occurred in 11.1% (*n* = 45) post-LT. Post-LT osteoporotic fractures occurred within a year in 16 recipients. Median time to first osteoporotic fracture was 1.5 years (IQR 0.9-2.9) and to major osteoporotic fracture 1.6 years (1.1-3.2).

The most common fracture site was vertebral. Nonosteoporotic fractures occurred in 10% of the total cohort (*n* = 42), predominantly in the foot and ankle (Table 2).

**Table 2:** Osteoporotic and Nonosteoporotic Fracture Sites Post-Transplant

Fracture site	No. of patients affected
Major osteoporotic fracture occurred in 43 recipients	
Vertebral fracture	32
Hip fracture	12
Distal radius fracture	3
Humerus	2
Other osteoporotic fracture occurred in 28 recipients	
Pelvis	10
Rib	22
Nonosteoporotic fracture occurred in 42 recipients	
Ankle	11
Metatarsal	19
Tarsal	8
Other^a^	13

Several patients in each category had multiple fractures.

Combination of hip, vertebral, and distal radius fracture; hip and vertebral fracture; hip and distal radius fracture each occurred in 1 individual.

a Other sites: phalanges (toes), fibula, ulnar styloid, tibial plateau, radial neck, scaphoid, and clavicle.

### Risk factors for osteoporotic fracture following LT

On univariate analyses, osteoporotic fracture pre-LT, age, female sex, prior glucocorticoid use, gym entry and exit-6MWT (distance in m/100), increased waitlist time, cumulative dose of ZA, and increased duration between receipt of ZA both pre- and post-LT and receipt of LT were associated with osteoporotic fractures, while CF conferred reduced risk. The same risk factors were observed for major osteoporotic fracture, with the exception of prior glucocorticoid use, and underlying cystic fibrosis (CF) was not protective (Supplement S2 and S3).

On multivariate analyses, predictors of osteoporotic fracture were pre-LT osteoporotic fracture, female sex, prior glucocorticoid use, and delayed receipt of ZA post-LT (Table 3). Patients who did not receive ZA post-LT were excluded from these analyses. Predictors of major osteoporotic fracture were pre-LT osteoporotic fracture, female sex, age at LT, and delayed receipt of ZA post-LT (Table 4). Prior glucocorticoid use did not reach statistical significance.

**Table 3:** Risk Factors for Post-Transplant Osteoporotic Fracture on Multivariate Survival Analysis With Death as a Competing Event (*n* = 336)

Factor	Hazard ratio (95% confidence interval)	*p*-value
Osteoporotic fracture pretransplant	2.25 (1.09-4.66)	0.026
Female sex	2.04 (1.06-3.90)	0.029
Age at transplantation	1.03 (0.98-1.07)	0.257
Glucocorticoid use pretransplant	1.98 (1.06-3.69)	0.029
History of smoking	1.12 (0.58-2.16)	0.726
Cystic fibrosis	0.40 (0.07-2.24)	0.285
Time post-transplant before ZA received (months)	1.04 (1.01-1.07)	0.002
Total number of infusions of ZA	1.15 (0.98-1.35)	0.085
6MWT at gym exit (m/100)	1.02 (0.78-1.35)	0.862
Duration on waiting list (months)	1.00 (0.96-1.05)	0.938

Abbreviations: 6MWT, 6-minute walk test; ZA, zoledronic acid.

**Table 4:** Risk Factors for Post-Transplant Major Osteoporotic Fracture on Multivariate Survival Analysis With Death as a Competing Event (*n* = 336)

Factor	Hazard ratio (95% confidence interval)	*p*-value
Osteoporotic fracture pretransplant	2.70 (1.17-6.24)	0.017
Female sex	2.24 (1.05-4.76)	0.033
Age at transplantation	1.08 (1.04-1.12)	<0.0001
Glucocorticoid use pretransplant	2.03 (0.93-4.46)	0.071
Time post-transplant before ZA received (months)	1.04 (1.02-1.07)	0.001
History of smoking	1.35 (0.64-2.86)	0.422
6MWT at gym exit (m/100)	1.21 (0.91-1.60)	0.189
Duration on waiting list (months)	1.01 (0.97-1.04)	0.735

Abbreviations: 6MWT, 6-minute walk test; ZA, zoledronic acid.

Of note, for each month’s delay in receiving ZA following LT, there was 4% increased risk of osteoporotic fracture. A 6- or 12-month delay increased risk by 27% (95%CI 17%-37%) and 60% (38%-87%), respectively. For major osteoporotic fracture, a 6- or 12-month delay in receipt of ZA increased risk by 29% (95%CI 11%-49%) and 65% (22%-123%), respectively. A sensitivity analysis using propensity matching showed 7% increased risk for osteoporotic fracture (HR: 1.07, 95%CI 1.02-1.12; *p* = 0.002) and 6% increased risk for major osteoporotic fracture (HR: 1.06, 95%CI 1.00-1.13; *p* = 0.033) for each month’s delay in receiving ZA post-LT.

### Antiresorptive therapy

Pre-LT, antiresorptive therapy was received by 47% (189/405) (Table 5). Median cumulative dose of ZA pre-LT was 4 mg (range 2-32, IQR 4-8) (Table 6). Female sex and longer duration on the LT waitlist were significantly associated with the administration of intravenous ZA pre-LT (Supplement S4). Median time on LT waitlist was 149 days (IQR 72-252) in those who received pre-LT ZA, and 57 days (IQR 23-141) in those who did not (*p* < 0.001).

**Table 5:** Use of Antiresorptive Therapy in Lung Transplantation Recipients Pre- and Post-transplantation and Associated Treatment-related Adverse Effects

Before lung transplantation	*n* (% of study cohort)
Received any antiresorptive therapy	189 (46.7%)
Received ZA	164 (40.5%)
Received ZA monotherapy	143 (35.3%)
Received only non-ZA agents	25 (6.2%)
	
Post lung transplantation	*n* (% of study cohort)
Received any antiresorptive therapy	360 (88.9%)
Received ZA	346 (85.4%)
Received ZA monotherapy	315 (77.8%)
Received only non-ZA agents	14 (3.5%)
	
ZA treatment-related adverse effect	*n* (% of those who received ZA) *n* = 359
Acute phase reaction	42 (11.7%)
Atypical femoral fracture^a^	2 (0.6%)
Osteonecrosis of the jaw	1 (0.3%)

a Three fractures in two patients.

**Table 6:** Associations Between Zoledronic Acid Use and Survival

Variable	Median (25-75th centile) or *n* (%)	Hazard ratio (95% confidence interval)	*p*-value
Pretransplant number of ZA infusions	0 (0-1)	1.06 (0.90-1.24)	0.461
Pretransplant cumulative dose of ZA (mg)	0 (0-4)	1.02 (0.98-1.05)	0.399
Post-transplant number of ZA infusions	3 (1-4)	0.67 (0.60-0.74)	<0.0001
Post-transplant cumulative dose of ZA (mg)	9 (4-16)	0.91 (0.89-0.94)	<0.0001
Total number of ZA infusions	3 (2-4)	0.77 (0.70-0.84)	<0.0001
Receipt of ZA at any time	359 (88.6%)	0.29 (0.19-0.44)	<0.0001
ZA received according to protocol	88 (21.7%)	0.70 (0.45-1.08)	0.101
At least 3 ZA infusions received	248 (61.2%)	0.48 (0.35-0.67)	<0.0001

Abbreviation: ZA, zoledronic acid.

Continuous data are presented here as median (25-75th centile) and categorical data as *n* (%).

Following LT, antiresorptive therapy was received by 89% (360/405) (Supplement S5). Most received ZA (346/360), median 3 doses (range 1-8, IQR 2-4), cumulative dose 12 mg (range 2-32, IQR 8-16). Median time to first dose ZA post-LT was 5 months (IQR 2-9).

At least 1 antiresorptive treatment was received both pre- and post-LT in 189 (47%), with 39% (*n* = 159) receiving ZA at both times. ZA was received by 89% (*n* = 359) either pre- or post-LT but only 22% (*n* = 88) received ZA as per protocol (Supplement S1) (i.e., 1 dose pre- and ≥2 doses post-LT). Specific antiresorptive therapy use is shown in Supplement S5.

Neither receipt of ZA ever, nor cumulative dose of ZA, was associated with fracture risk on univariate analyses. There was also no association between ZA use according to protocol and osteoporotic fracture (HR 0.61 (0.29-1.25), *p* = 0.17) or major osteoporotic fracture (HR 0.46 (0.18-1.19), *p* = 0.10).

Receipt of ZA ever, total number of ZA infusions, number of infusions post-LT, and having >2 infusions post-LT were all associated with improved survival on univariate analyses (Table 6). However, there was no survival benefit in those who received ZA per protocol (*n* = 88, 28.4% mortality) compared with those who did not (*n* = 317, 38.2% mortality, HR 0.7 (0.45-1.08), *p* = 0.10).

Fracture pre- or post-LT was not associated with mortality (pre-LT fracture HR 1.19 (0.74-1.93), *p* = 0.47, post-LT fracture HR 0.77 (0.47-1.25), *p* = 0.28). There was no significant difference in the proportion of LT recipients who received ZA pre- or post-LT based on their post-LT fracture status (Supplement S6).

Treatment-related adverse effects were uncommon (Table 5). Two individuals sustained atypical femoral fractures (AFF). The first had 6 infusions of ZA (25 mg) over 4 years. Bilateral AFFs occurred 5 years post-LT. The second had a complex history of osteoporosis with vertebral and femoral fractures preceding LT, brief treatment with risedronate, denosumab for 2 years (ceased 1-year pre-LT), then 1 ZA infusion post-LT. Both subsequently received teriparatide. There was 1 case of jaw osteonecrosis in an individual who received denosumab pre-LT and 4 doses of ZA post-LT.

## Discussion

### Main findings

This contemporary study followed 405 adult LT recipients for 5 years at an institution with protocolized antiresorptive therapy. Almost half received ZA before transplantation and over 85% received it after, with a median time to first dose of 5 months. Fifteen percent (*n* = 60) sustained a post-LT osteoporotic fracture after a median of 1.5 years. Cumulative incidence of post-LT osteoporotic fracture with death as a competing variable was 12% at 3 years and 14% at 5 years. Risk factors for osteoporotic fracture following LT were prior osteoporotic fracture, female sex, glucocorticoid use pre-LT, and longer time to first dose ZA post-LT. Risk factors for major osteoporotic fracture were prior osteoporotic fracture, female sex, increased age at transplantation, and longer time to first dose ZA post-LT. Prior glucocorticoid use was not statistically significant in the latter, likely due to reduced power consequent upon fewer major fractures.

This study is unique in that detailed temporal analyses for fractures and death were performed. Specifically, death was included as a competing variable when calculating fracture incidence and risk factors. The risk of fracture is estimated more accurately by this analysis since it may be underestimated if death rates are high following LT, particularly if there are more deaths than fractures.

### Comparison to other studies

The prevalence of osteoporotic fractures post-LT in our cohort was lower than reported in historical studies.^21^, ^22^, ^23^, ^24^, ^25^ In studies with antiresorptive therapies post-LT, prevalence of osteoporotic fractures was 15% to 35%.^26^, ^27^, ^28^ Grassi et al evaluated 117 LT recipients of whom 84% received bone preservation therapies and reported an incidence of 15% over 2 years.^27^ In 131 LT recipients with cystic fibrosis, most of whom received oral bisphosphonates, a fracture prevalence of 21% was noted.^26^ Most recently, a fracture prevalence of 35% in 284 LT recipients over 5 years was reported.^28^ These studies did not assess cumulative fracture incidence. The lower fracture incidence in our study may relate to increased use of protocolized bisphosphonate therapy. Use of ZA or cumulative dose was not associated with reduced fracture risk, most likely because almost all recipients received it.

Risk of osteoporotic fracture is highest in the first year after LT, with prevalence of 9.5% to 37%.^10^, ^11^, ^27^, ^29^ Our 1-year prevalence was 4%. Although we excluded 16 patients who died within 6 months of transplantation to avoid bias related to perioperative frailty, this was unlikely to explain the lower prevalence. We postulate that routine use of ZA before and after LT may have provided bone protection during this high-risk period. Additional explanations may include our center’s requirement for LT recipients to undertake a 3-month, outpatient rehabilitation program under the care of a senior physiotherapist. This includes 3 gym-based sessions per week that include strength and balance training. However, ours is not the only center to offer such a program. Other explanations such as reduced glucocorticoid exposure in our cohort are unlikely as we do not have a more rapid wean of prednisolone than other centers.

### Predictors of osteoporotic fracture after transplantation

Prior osteoporotic fracture more than doubled the risk of post-LT fracture. Individuals with osteoporotic fractures pre-LT represent a high-risk group with significant bone loss during the waiting period.^26^ Vertebral osteoporosis is found in 25% to 50% in LT candidates.^21^, ^30^ Propensity to fracture may indicate additional genetic and/or physiological factors driving bone fragility. Factors such as smoking and chronic glucocorticoids may exacerbate bone loss,^21^ but sample size may have limited power to detect a significant association.

Pre-LT glucocorticoid use increased post-LT osteoporotic fracture risk, likely due to the cumulative effect of glucocorticoids on BMD before and immediately post-LT. High-dose glucocorticoids, the cornerstone of immunosuppression, are linked to rapid bone loss within the first 6 months following LT which continues for at least another year thereafter.^6^, ^11^, ^17^, ^22^, ^31^

The post-LT period of accelerated BMD loss is critical for intervention. Our earlier study showed that ZA administration within 6 months of LT was associated with reduced BMD loss.^32^ Grassi et al also reported that antiresorptive use before or at LT either averted loss or improved lumbar spine and femoral neck BMD over 2 years.^27^ The current study found timing of ZA post-LT to significantly predict both time to first osteoporotic fracture and first major osteoporotic fracture, suggesting that early ZA administration reduces bone loss. Overall use of ZA was not a predictor. However, this might be due to high ZA use, and the exclusion of those who did not receive it from multivariate analyses because time to first dose was an included variable. Despite the lack of association between ZA and fracture, its use was associated with reduced mortality as was observed previously in postmenopausal osteoporosis.^33^ Although beyond the scope of this study, assessing survival and mortality risk factors is an important subject for future study.

### Treatment-related adverse effects

The rate of acute phase reactions was 11.7%, which was lower than the 42.4% reported in postmenopausal women in the HORIZON Trial^33^ and other studies.^34^, ^35^, ^36^ This is likely attributable to using premedications (paracetamol, glucocorticoids), slow infusion rates, and reduced ZA dose for low BMI and renal dysfunction. AFFs were rare (3 in 2 recipients), with 1 case occurring with multiple antiresorptive doses and the other in severe pre-existing osteoporosis.

### Implementation of antiresorptive therapy protocol

The lower rate of pre- versus post-LT ZA in our cohort was likely attributable to the short time on the LT waitlist combined with delayed endocrinology assessment given our large catchment area encompassing 3 adjacent states. Addressing peritransplantation management barriers requires improved collaboration between tertiary and community services. The capacity of our service to provide express access for same-day ZA increased individual adherence to the protocolized schedule, although this was impacted by COVID-19.

Endocrinologist involvement in the multidisciplinary lung transplant team may have contributed to protocol adherence and greater focus on bone health. Attention to modifiable factors such as vitamin D and calcium supplementation, nutritional support, and biochemical monitoring to minimize bone toxicity may have also impacted results. Given the time period of this study, use of anabolic agents was limited (*n* = 16) but robust evidence indicates that teriparatide is superior to oral bisphosphonates in glucocorticoid-induced osteoporosis, with significant vertebral gains^37^, ^38^, ^39^, ^40^ and improved lung function.^41^

### Strengths and limitations

Study strengths include a large sample size and consistent approaches to immunosuppression, antiresorptive therapy, and post-LT rehabilitation. Clinically relevant fractures were end-points rather than BMD, a surrogate marker. Careful examination of individual medical records determining the date and site of fractures and osteoporosis treatments allowed detailed temporal analyses. Using death as a competing risk allowed for accurate representation of the probability of fracture in surviving patients. Importantly, our study adds to the limited literature on long-term fractures in LT recipients following bone preservation therapies.

Limitations are the retrospective, single-center design, although our cohort’s demographics are similar to previously published national and international trends^42^, ^43^, ^44^ and the population prevalence of osteoporosis in the Australian population is similar to that in the US and UK.^45^, ^46^, ^47^

We did not compare this cohort to a historical control because of differences between the groups. Since 2012, our transplant recipients are an average of 5 years older, with more interstitial lung disease and less CF. The immunosuppressive regimen has also changed, with a switch from azathioprine and cyclosporine to mycophenolate and tacrolimus. We believe these differences would confound between-group comparisons.

We could not comment on the specific benefits of ZA on bone health, because of the high take-up. However, a placebo-controlled, randomized trial of antiresorptive effects in LT recipients would now be unethical.

## Conclusion

In this large retrospective study of LT recipients with protocolized ZA use, cumulative incidence of osteoporotic fracture was lower than historical reports. Prior osteoporotic fracture, delayed timing of ZA administration post-LT, female sex, and prior glucocorticoid use were predictors of osteoporotic fracture post-LT. Intravenous ZA was well tolerated and our findings support its ongoing use in adult LT recipients.

## Author contributions

Elisabeth Ng: investigation, data curation, validation, writing—original draft and review, project administration; Shanal Kumar: investigation, writing—original draft; Eldho Paul: methodology, formal analysis; Daniel Bennett: investigation; Luisa Rosi: investigation; Louise Fuller: investigation; Lauren Chiu: investigation; Shoshana Sztal-Mazer: writing—review; Steven Ivulich: investigation; Greg Snell: writing—review and editing, supervision; Leon Bach: writing—review and editing, supervision; Kathryn Hackman: conceptualization, methodology, investigation, writing—review and editing, project administration.

## Disclosure statement

The authors declare that they have no known competing financial interests or personal relationships that could have appeared to influence the work reported in this paper.

No funding was received for this study.

## Data Availability

Ethics approval for this project does not extend to sharing of data for this study. Specific data requests should be made to the corresponding author.

## Footnotes

Appendix A Supplementary data associated with this article can be found in the online version at doi:10.1016/j.jhlto.2024.100182.
