# Supplementary material for: Incidence, prevalence, and predictors of osteoporotic fracture in adult lung transplant recipients
Source: JHLT Open. 2024 Nov 20;7:100182. doi: 10.1016/j.jhlto.2024.100182 (PMC11935502; doi:10.1016/j.jhlto.2024.100182)
Supplement: Supplementary file 1 — Supplementary material [file mmc1.docx]

# Supplementary Material

# ***Incidence, prevalence and predictors of osteoporotic fracture in adult lung transplant recipients (Ng et al)***

S1: Alfred Health protocol for management of lung transplant candidates instituted in 2012

S2: Univariate survival analysis with death as a competing risk for time to first osteoporotic fracture

S3: Univariate survival analysis with death as a competing risk for time to first major osteoporotic fracture

S4: Associations between administration of pre-transplantation zoledronic acid and recipient age, sex and transplant waiting time

S5: Specific antiresorptive therapies according to pre- or post-transplantation status

S6: Zoledronic acid infusion pre- and post-transplantation according to osteoporotic fracture timing

**S1: Alfred Health protocol for management of lung transplant candidates instituted in 2012**

This protocol has been updated since the original version was published in 2012.


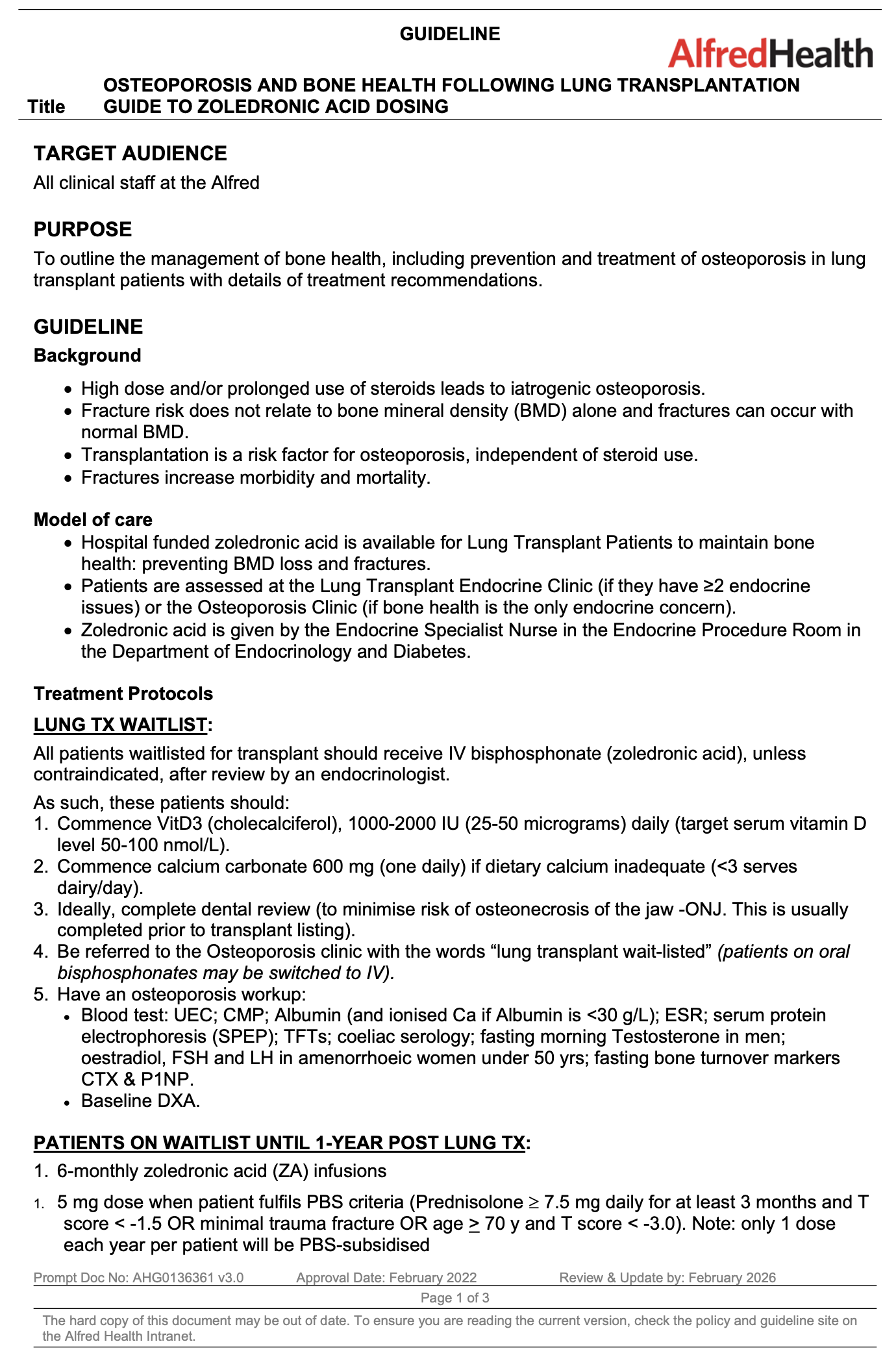


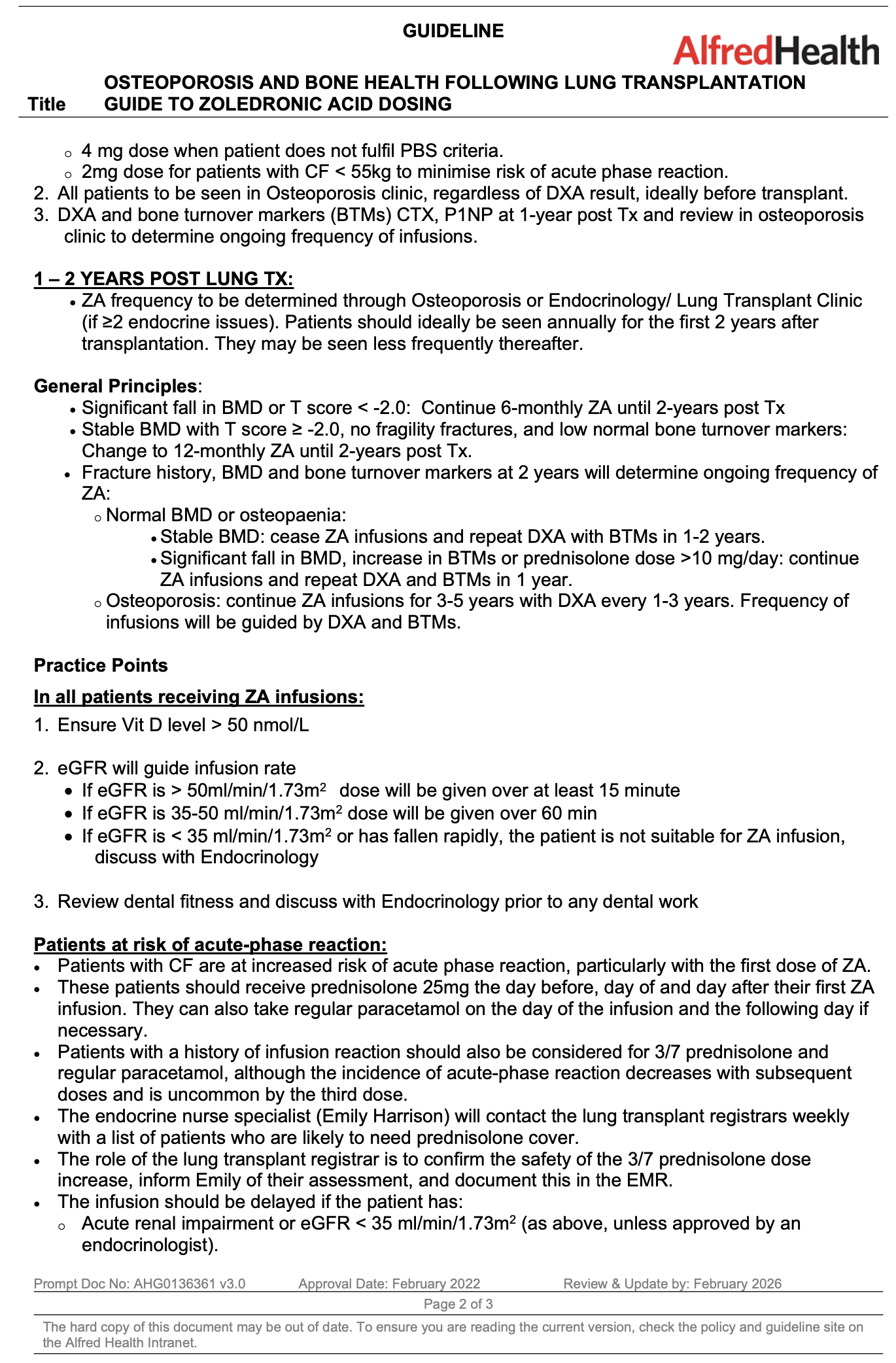


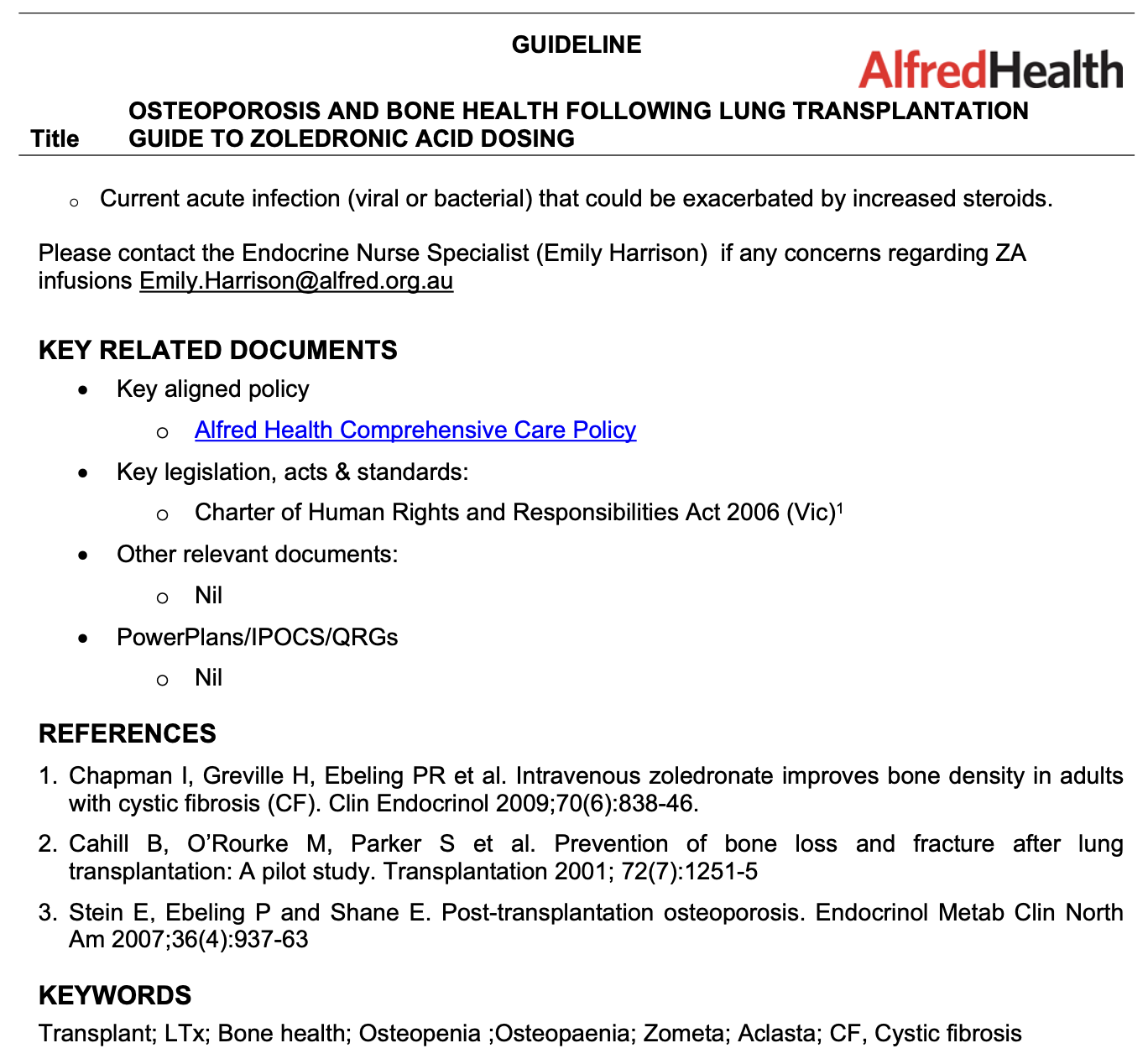


**S2: Univariate survival analysis with death as a competing risk for time to first osteoporotic fracture**

| **Factor** | **Hazard Ratio (95% confidence interval)** | **p-value** |
| --- | --- | --- |
| Osteoporotic fracture pre-LT | 2.55 (1.36 - 4.79) | 0.003 |
| Female sex | 2.20 (1.30 - 3.74) | 0.003 |
| History of smoking | 1.71 (0.97 – 2.99) | 0.057 |
| Age at transplantation | 1.03 (1.01 - 1.06) | 0.007 |
| Glucocorticoid use pre-LT | 1.81 (1.03 – 3.17) | 0.036 |
| Methylprednisolone administration pre-fracture (n=13) | 0.52 (0.24 – 1.14) | 0.09 |
| Methylprednisolone administration post-LT | 0.84 (0.50 – 1.41) | 0.49 |
| Doses of methylprednisolone pre- or post-LT | 0.93 (0.70 – 1.23) | 0.59 |
| Underlying lung disease |  |  |
| Cystic fibrosis | 0.29 (0.09 – 0.95) | 0.037 |
| Obstructive lung disease | 1.35 (0.80 – 2.26) | 0.249 |
| Interstitial lung disease | 1.22 (0.70 – 2.12) | 0.476 |
| Pulmonary hypertension | 0.49 (0.12 – 2.07) | 0.325 |
| Bronchiectasis | 1.69 (0.51 – 5.65) | 0.381 |
| Last dose ZA prior to Tx (months) | 1.01 (0.95 - 1.06) | 0.799 |
| Time to first dose ZA post-LT (months) | 1.02 (1.00-1.05) | 0.049 |
| Time to last dose ZA post-LT (months) | 1.01 (1.00 – 1.02) | 0.016 |
| Total number of infusions of ZA | 1.13 (0.99 – 1.27) | 0.055 |
| Time on waiting list | 1.00 (1.00 – 1.00) | 0.002 |
| 6MWT at gym entry (metres/100) | 0.81 (0.66 – 0.98) | 0.026 |
| 6MWT at gym exit (metres/100) | 0.84 (0.74 – 0.95) | 0.006 |
| 6MWT at gym exit (tertiles) | 0.65 (0.44 – 0.98) | 0.034 |
| Body mass index (kg/m^2^) | 1.02 (0.96 – 1.09) | 0.528 |

Abbreviations – 6MWT: 6-minute walk test; LT: lung transplantation; ZA: zoledronic acid

**S3: Univariate survival analysis with death as a competing risk for time to first major osteoporotic fracture**

| **Factor** | **Hazard Ratio (95% confidence interval)** | **p-value** |
| --- | --- | --- |
| Osteoporotic fracture pre-LT | 2.85 (1.46 - 5.56) | 0.002 |
| Female sex | 2.38 (1.29 - 4.40) | 0.005 |
| History of smoking | 1.78 (0.93 – 3.41) | 0.077 |
| Age at transplantation | 1.05 (1.02 - 1.08) | 0.001 |
| Glucocorticoid use pre-transplant | 1.87 (0.97 – 3.61) | 0.057 |
| Methylprednisolone administration post-LT (n= 26) | 0.72 (0.39 – 1.32) | 0.282 |
| Doses of methylprednisolone post-LT | 0.89 (0.61 – 1.28) | 0.511 |
| Underlying lung disease |  |  |
| Cystic fibrosis | 0.26 (0.06 – 1.13) | 0.067 |
| Obstructive lung disease | 1.34 (0.74 – 2.44) | 0.327 |
| Interstitial lung disease | 1.45 (0.76 – 2.75) | 0.247 |
| Pulmonary hypertension | 0.34 (0.05 – 2.63) | 0.294 |
| Bronchiectasis | 0.69 (0.10 – 4.99) | 0.708 |
| Last dose ZA prior to LT (months) | 1.03 (0.98 – 1.08) | 0.28 |
| Time to first dose ZA post-LT (months) | 1.03 (1.00 - 1.05) | 0.027 |
| Time to last dose ZA post-LT (months) | 1.02 (1.01 - 1.03) | 0.004 |
| Total number of infusions of ZA | 1.05 (0.92 – 1.20) | 0.440 |
| Time on waiting list (months) | 1.04 (1.01 – 1.07) | 0.016 |
| 6MWT at gym entry (metres/100) | 0.72 (0.58 - 0.89) | 0.002 |
| 6MWT at gym exit (metres/100) | 0.85 (0.74 - 0.97) | 0.016 |
| 6MWT at gym exit (tertiles) | 0.57 (0.37 - 0.88) | 0.010 |
| Body mass index (kg/m^2^) | 1.00 (0.93 – 1.07) | 0.972 |

Abbreviations – 6MWT: 6-minute walk test; LT: lung transplantation; m: metres; ZA: zoledronic acid

**S4: Associations between administration of pre-transplantation zoledronic acid and recipient age, sex and transplant waiting time**

|  | **Zoledronic acid received pre-transplantation (n=163)** | **No zoledronic acid received pre-transplantation (n=242)** | **P value** |
| --- | --- | --- | --- |
| **Age (years)** | 60 (52-65) | 59 (46-64) | 0.07 |
| **Female sex** | 78 (47.9%) | 90 (37.2%) | **0.03** |
| **Waiting time (days)** | 149 (72-252) | 57 (23-141) | **<0.001** |

Continuous data is presented here as median (25-75 centile) and categorical data as n (%).

**S5: Specific antiresorptive therapies according to pre- or post-transplantation status**

|  | **Pre-transplantation**  **n(%)** | **Post-transplantation**  **n(%)** |
| --- | --- | --- |
| Any treatment | 190 (46.7%) | 360 (88.9%) |
| Zoledronic acid | 165 (40.7)  (150 received ZA alone) | 346 (85.4)  (315 received ZA alone) |
| Alendronate | 24 (5.9) | 1 (0.25) |
| Risedronate | 15 (3.7) | 13 (3.2) |
| Denosumab | 12 (3.0) | 24 (5.9) |
| Teriparatide | 1 (0.25) | 15 (3.7) |
| Other | 2 (0.49) | 1 (0.25) |
| Combination of 2 therapies | 27 (6.7) | 34 (8.4) |
| Combination of 3 therapies | 1 (0.25) | 6 (1.5) |

**S6: Zoledronic acid infusion pre- and post-transplantation according to osteoporotic fracture timing**

|  | Pre-transplantation | | | Post-transplantation | | |
| --- | --- | --- | --- | --- | --- | --- |
|  | **Received ZA, n (%)*** | **Number of ZA infusions** | **Cumulative dose (mg)** | **Received ZA, n (%)***^#^* | **Number of ZA infusions** | **Cumulative dose (mg)** |
| **Pre-transplant fracture**  **(n=37)** | 19 (51.4%) | 2 (1-3) | 8 (5-14) | 33 (89.2%) | 2 (1-4) | 9 (4-16) |
| **No pre-transplant fracture**  **(n=368)** | 145 (39.4%) | 1 (1-2) | 4 (4-6) | 313 (85.1%) | 3 (2-4) | 12 (8-16) |
| **Post-transplant fracture**  **(n=64)** | 27  (42.2%) | 2 (1-3) | 7 (4-12) | 54 (84.4%) | 3 (2-4) | 12 (9-18) |
| **No post-transplant fracture**  **(n=341)** | 137  (40.2%) | 1 (1-2) | 4 (4-6) | 292 (85.6%) | 3 (2-4) | 12 (8-16) |

Continuous data is presented here as median (25-75 centile) and categorical data as n (%).

Abbreviations – ZA: zoledronic acid

*NB: *There was no significant difference in rates of ZA infusions between lung transplantation (LT) recipients who had a pre-LT osteoporotic fracture compared to those who did not (p=0.16).*

*# There was no significant difference in LT recipients post-transplant who received ZA based on osteoporotic fracture status pre-transplant (p = 0.50), nor was there a difference in those who received it based on osteoporotic fracture status post-transplant (p = 0.50).*
